# PROTAC: a revolutionary technology propelling small molecule drugs into the next golden age

**DOI:** 10.3389/fonc.2025.1676414

**Published:** 2025-10-27

**Authors:** Jiale Cai, Chen Chen, Jiayue Wang, Xinmeng Zhang, Yuqiu Cui, Qunshan Zhu, Haibo Sun

**Affiliations:** ^1^ Institute of Translational Medicine, Medical College, Yangzhou University, Yangzhou, Jiangsu, China; ^2^ Jiangsu Key Laboratory of Experimental & Translational Non-Coding RNA Research, Yangzhou University, Yangzhou, Jiangsu, China; ^3^ Department of Gastrointestinal Surgery, Jiangdu People’s Hospital Afliated to Yangzhou University, Yangzhou, China

**Keywords:** PROTAC, protein degradation, targeted therapy, cancer, clinical progress

## Abstract

Proteolysis Targeting Chimera (PROTAC) is a heterobifunctional molecule comprising three core components: a target protein ligand (typically a small-molecule inhibitor), a linker, and an E3 ubiquitin ligase ligand. By harnessing the specificity of the endogenous ubiquitin-proteasome system (UPS), PROTACs induce ubiquitination and subsequent degradation of target proteins. This technology constitutes an advanced therapeutic strategy for selective protein degradation, thereby expanding the horizons of drug design. Its significant therapeutic potential extends to treating cancers, viral infections (e.g., HIV and SARS-CoV-2), and chronic diseases. Recent clinical studies on compounds such as ARV-471 have yielded encouraging results, validating the efficacy of this approach. Over the past decade, PROTAC technology has garnered widespread attention in biomedicine for its promise in developing novel targeted therapies. This review will elucidate the broad therapeutic prospects and future challenges of PROTACs by detailing their mechanism of action, recent advances, progress in targeted therapy research, and current clinical trial landscape.

## 1 Introduction to the principles of PROTAC

### 1.1 The ubiquitin-proteasome system

The ubiquitin-proteasome system (UPS) serves as the primary proteolytic pathway for regulated protein degradation. It tightly governs numerous cellular processes in eukaryotic cells—including DNA repair, stress response, and cell proliferation—by mediating the selective and specific degradation of proteins to control cellular protein levels (1). Essential components of the UPS include ubiquitin, ubiquitin-activating enzymes (E1s), ubiquitin-conjugating enzymes (E2s), ubiquitin ligases (E3s), deubiquitinating enzymes (DUBs), and the 26S proteasome (2, 3). Ubiquitin conjugation to substrate proteins occurs via a multi-step, largely reversible enzymatic cascade involving E1, E2, and E3 enzymes. This process comprises three principal steps: First, E1 activates ubiquitin using energy derived from ATP hydrolysis and transfers it to an E2 enzyme. Subsequently, an E3 ligase facilitates the transfer of the activated ubiquitin from the E2 to a specific substrate protein. Once at least four ubiquitin molecules are conjugated to the substrate (4, 5), a polyubiquitin chain is formed. Finally, the polyubiquitinated substrate is recognized and hydrolyzed by the 26S proteasome. Following target protein degradation, ubiquitin recycling enzymes disassemble the polyubiquitin chain into monomeric ubiquitin molecules, allowing for ubiquitin reuse.

### 1.2 Mechanism of action of PROTAC

**Figure 1:**
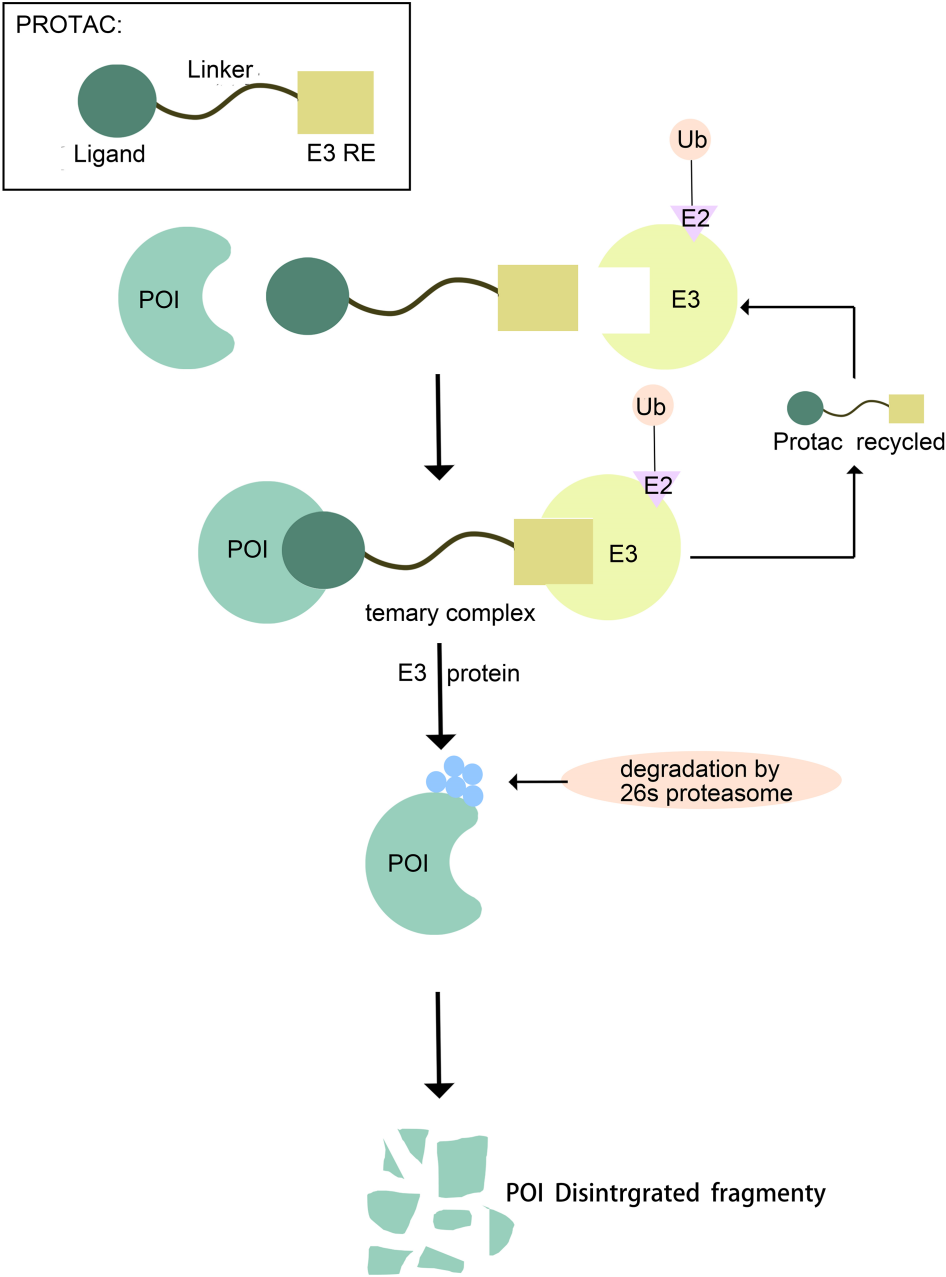
Mechanism of Protein Degradation Mediated by PROTAC.

A PROTAC (Proteolysis-Targeting Chimera) molecule comprises three core components: a ligand for the target protein (typically a small-molecule inhibitor), a linker, and a ligand for an E3 ubiquitin ligase. These components are covalently linked, often via linkers containing 5–15 carbon atoms or other atoms (6). Functionally, the target protein ligand selectively binds the protein of interest (POI), the E3 ligase ligand recruits an E3 ubiquitin ligase, and the linker connects these two ligands, effectively bridging the POI and the E3 ligase (7). This interaction facilitates the formation of a POI-PROTAC-E3 ligase ternary complex (8, 9). Within this complex, the E3 ligase ubiquitinates the POI, marking it for recognition and subsequent degradation by the 26S proteasome (10). Following degradation, the PROTAC molecule dissociates and can participate in further degradation cycles, enabling its intracellular recycling (11). Thus, PROTACs represent a technology that utilizes the intracellular ubiquitin—proteasome system (UPS) to achieve targeted protein degradation (12). (**Figure 1**).

More than two decades after Craig M. Crews pioneered the first PROTAC in 2001, this technology has achieved significant breakthroughs. Unlike traditional drugs, PROTACs bypass the need for direct binding to an active site by inducing target protein degradation upon engagement. This mechanism enables PROTACs to overcome targets traditionally deemed “undruggable” due to the absence of a suitable active-site binding pocket. Secondly, while conventional inhibitors require sustained high concentrations to saturate and inhibit their targets, PROTACs function catalytically: they induce target degradation, dissociate from the complex, and can then catalyze multiple subsequent degradation cycles. Consequently, PROTACs achieve their effects at significantly lower concentrations. Finally, because PROTACs act through target degradation—a mechanism independent of the active site—they can effectively overcome resistance driven by target overexpression or mutations within the active site (13).

## 2 Development of PROTAC

**Figure 2:**
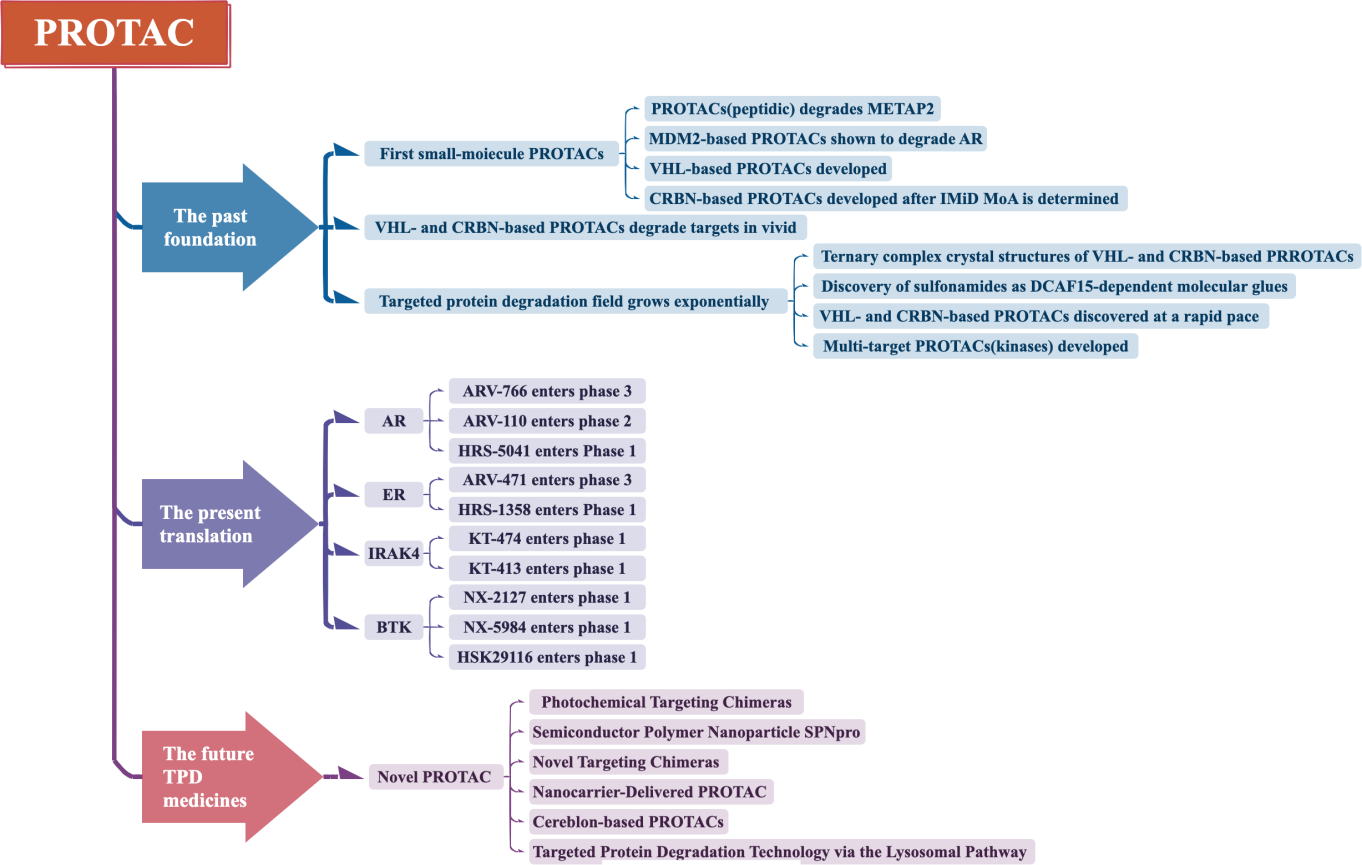
Development of PROTAC.

PROTAC technology has currently undergone three generations of development (**Figure 2**).

### 2.1 First generation—peptide-based PROTAC

The Crews team developed the first peptide-based PROTAC-1 molecule in 2001 (14). This design aimed to promote the degradation of methionine aminopeptidase-2(MetAP-2). PROTAC-1 is composed of three covalently linked components. One domain containing a phosphorylated IκBα peptide recognized by the Skp1-Cullin-F-box complex (SCF, an E3 ligase that initiates protein ubiquitination and UPS degradation).one domain containing ovalicin (a MetAP-2 inhibitor), and a linker that connects these two domains. This study elucidated the original concept of Protein Degrader Targeting Chimeras (PROTAC), namely, inducing the degradation of the target protein (POI) through UPS in live cells.

Based on this principle, researchers subsequently invented various peptide-based PROTACs to eliminate overexpressed proteins in diseases. These proteins include androgen receptor (AR), estrogen receptor (ER), FK506-binding protein (FKBP12), and aryl hydrocarbon receptor (AHR), among others (15–18). The discovery of these peptide-based PROTACs provides new insights for developing novel therapeutic approaches and drugs. By selecting appropriate peptide segments and target proteins, researchers can design PROTACs with high selectivity and potency, promoting the degradation of disease-related proteins. This holds the promise of significant advancements in the field of drug development.

### 2.2 Second generation—small molecule-based PROTAC

#### 2.2.1 Different E3 ligase PROTACs

In light of the significant lipophilicity demonstrated by certain small molecules, the Crews team pioneered the development of the first small molecule-based PROTAC in 2008. The primary goal of this design was to effectively degrade the androgen receptor (AR) in cancer cells. This PROTAC employed the MDM2-p53 protein-protein interaction (PPI) inhibitor nutlin-3a as the E3 ligase recruiter. Additionally, a selective androgen receptor modulator (SARM), a derivative of flutamide, served as the AR-targeting ligand. The two components were linked by a PEG-based linker (19). Through this mechanism, nutlin-3a facilitated the recruitment of the ubiquitin ligase complex, leading to the binding of the target protein AR and its subsequent ubiquitination. Simultaneously, SARM also bound to AR, putting it in a state conducive to ubiquitination and degradation. With the help of the linker, these two components formed a small molecule PROTAC, achieving selective degradation of AR.

Through continuous development, in 2010, Itoh and colleagues successfully synthesized another type of PROTAC molecule using the chemical compound methyl bestatin. The molecule facilitated the degradation of the target protein by recruiting the E3 ligase inhibitor of apoptosis protein (IAP). In an effort to improve both its efficacy and selectivity toward the target, researchers incorporated small molecules that exhibit high affinity and specificity. For instance, imide compounds were utilized, which are capable of recruiting the E3 ligase cereblon (CRBN) (20). Alternatively, compounds with a strong binding affinity for the E3 ligase VHL-1 were also explored (21). These small molecules were incorporated into PROTACs to regulate the degradation of various cancer-related targets, such as Ikaros family zinc finger proteins 1/3 (IKZF1/3) and estrogen-related receptor alpha (ERRα).

By adjusting the structure and linkage of small molecule components, it is possible to design small molecule PROTACs with high selectivity and potency, allowing for precise control of specific proteins. This research advancement provides a new direction for developing novel therapeutic approaches and drugs, offering the potential for significant breakthroughs in the treatment of diseases such as cancer.

#### 2.2.2 Different Target PROTACs

##### 2.2.2.1 PROTAC targeting epigenetics

Epigenetics are the reversible processes by which gene expression changes occur without changing the DNA nucleotide sequence (22). PROTACs hold great promise in epigenetic treatment by targeting the critical regulators of gene expression, including histone modifiers, chromatin remodelers, and transcriptional regulators. These proteins play crucial roles in many diseases, especially in cancers and neurodegenerative diseases, and thus PROTACs provide a very promising therapeutic approach (23).

###### 2.2.2.1.1 EZH2

An adaptor subunit of the Polycomb Repressive Complex 2 (PRC2) that functionally plays a part in gene silencing by means of histone methylation. EZH2 downregulation may reverse cancer epigenetic silencing, particularly hematologic and solid malignancies (24).

###### 2.2.2.1.2 HDAC6

Histone deacetylase that regulates acetylation. Downregulation of HDAC6 by PROTAC could restore acetylation homeostasis with probable therapeutic application for cancer, neurodegenerative disorders, and fibrosis (25).

###### 2.2.2.1.3 p300/CBP

Acetyltransferases responsible for the activation of genes (26). The proteolysis of such proteins by PROTACs may provide gene expression with precise control, and it may be utilized in cancer therapy, cardiovascular disease, and neurodegenerative diseases (27).

###### 2.2.2.1.4 BRD4

It’s a BET family protein, which is crucial for transcriptional regulation as it binds to acetylated histones or transcription factors and recruits P-TEFb for phosphorylation of RNA polymerase II to promote gene transcription (28). Excessively over-regulated BRD4 and other BET proteins disrupt chromatin remodeling and gene expression and induce various cancers (29). Specifically, the PROTAC dBET, designed by the Bradner lab, brings together JQ1 (BRD4 ligand) and thalidomide (CRBN ligand) to initiate ubiquitination and degradation of BRD4, offering a targeted treatment (30).

##### 2.2.2.2 PROTAC Targeting BTK

BTK is a non-receptor tyrosine kinase that is predominantly found in hematopoietic cells (31). It plays a central role in the signaling of the B-cell receptor (BCR) and is directly involved in B-cell tumor growth and development (32). Upon antigen binding to the BCR, BTK is translocated to the plasma membrane from the cytoplasm. It becomes activated and phosphorylated by SRC family kinases there. In addition, BTK can activate a number of signaling cascades including AKT, ERK, and NF-κB, which are involved in regulating cell survival and proliferation and hence enhance cell viability.

To inhibit the function of BTK, Gray and colleagues developed the first PROTAC molecule targeting BTK, known as D-04-015 (33). This molecule consists of pomalidomide and the ligand RN486 for BTK. D-04–015 can selectively degrade BTK, consequently inhibiting the proliferation of B-cell lymphoma TMD8 cells. This study provides a new possibility for treating BTK by selectively degrading it using PROTAC technology, effectively suppressing the growth of B-cell tumors. Further research and development in this area aim to optimize these BTK PROTAC molecules, enhancing their efficacy and selectivity, and providing more treatment options for B-cell-related diseases, particularly B-cell lymphoma.

##### 2.2.2.3 PROTACs Targeting KRAS

Proteins of the RAS gene family lack suitable small molecule binding pockets, rendering them challenging drug targets. Notably, KRAS mutations account for approximately 85% of RAS mutations. The KRAS^G12C^ mutation, a specific variant, can be targeted with the small molecule inhibitor MRTX849; however, prolonged use of this drug activates cellular compensatory mechanisms, leading to resistance. Researchers from the BOND study constructed a VHL-based PROTAC using MRTX849 (34), which rapidly and sustainably induces degradation of KRAS^G12C^, thereby inhibiting the MAPK signaling pathway. This innovation opens new avenues for treating KRAS-mutant malignancies. Currently, in various xenograft models of aggressive tumors harboring KRAS^G12D^ mutations—including pancreatic, colorectal, and non-small cell lung cancers—these PROTACs demonstrate potent antitumor activity, significantly reducing KRAS^G12D^ protein levels and blocking the extracellular signal-regulated kinase pathway, ultimately inducing tumor cell apoptosis (35).

##### 2.2.2.4 Targeting IRAK4

IRAK4 within the IRAK kinase family mediates pro-inflammatory cytokine production via the NF-κB and JNK signaling pathways. Loss of IRAK4 function can impair normal inflammatory responses, increasing susceptibility to severe bacterial infections, while hyperactivation may trigger autoimmune diseases.

KT-474 interacts with CRBN and IRAK4 to form a ternary complex. This interaction promotes the ubiquitination of IRAK4, leading to its degradation via the proteasomal pathway. As a result, downstream signaling is inhibited, thereby suppressing cell activation and the induction of cytokines mediated by TLR and IL-1R (36).

The IRAK4 structure includes a binding site for small molecule inhibitors; however, its activation of NF-κB and JNK pathways involves different mechanisms. NF-κB relies on its scaffold function, whereas JNK depends on phosphorylation, making comprehensive inhibition with small molecules challenging (37). IRAK4-targeted PROTACs can eliminate both enzymatic and non-enzymatic functions of IRAK4, and due to better cellular penetration in immune cells compared to solid tumors, they represent a promising new strategy for autoimmune disease therapy.

##### 2.2.2.5 PROTACs Targeting Tau Protein

One of the causes of Alzheimer’s disease is the hyperphosphorylation of Tau protein, which reduces its affinity for microtubule proteins, leading to neurofibrillary tangles (38). Current treatments mainly involve antisense oligonucleotides and monoclonal antibodies; however, antisense oligonucleotides also lower non-pathological Tau levels, and Tau gene knockout in some animal models results in cognitive and motor deficits. Monoclonal antibodies struggle to cross cell membranes, limiting their effectiveness to extracellular Tau pathology. PROTACs bring Tau proteins into close proximity with E3 ligases, promoting polyubiquitination and proteasome-mediated degradation. In cultured cells, PROTACs have been shown to effectively degrade Tau (39). In mouse models of Alzheimer’s disease (AD) and Tauopathies, Tau-targeting PROTACs lead to a reduction in both total Tau and phosphorylated Tau levels. Additionally, they help preserve dendritic arborization and enhance cognitive function (40). Some studies have successfully employed single-domain antibodies (sdAbs) as PROTAC targeting moieties to rapidly degrade various proteins (41), showing great potential in neurodegenerative disease research. Currently, PROTAC technology offers the possibility of inhibiting Tau aggregation by inducing dephosphorylation (42), holding promise as a therapeutic approach for Alzheimer’s disease and other neurodegenerative disorders.

### 2.3 Third generation—novel PROTAC

#### 2.3.1 Photochemical targeting chimeras

When PROTAC is systemically administered, it may have effects on both tumor and normal cells, leading to off-target effects and non-specific toxicity. To address this issue, researchers have proposed Photochemical Targeting Chimeras (PHOTAC) (43). PHOTACs are composed of a protective group and a substituent group removable by visible light, where the removable substituent group can be a nitrobenzyl-ligand-oxo-carbonyl or a visible light-cleavable substituent, such as azobenzene (44). These molecules are inactive in the dark, but under UVA or visible light exposure, the removable substituent group dissociates from the visible light-cleavable substituent undergoes a conformational change, forming an active protective group and triggering the degradation of the target protein.

Compared to traditional PROTACs, PHOTACs exhibit more controlled degradation effects on both temporal and spatial scales, potentially reducing adverse toxicities associated with PROTACs. Recently, various types of PHOTACs have been reported, successfully degrading numerous target proteins such as BRD4, FKBP12, IKZF1/3, ALK, and BTK, among others (45). The research on these photochemical targeting chimeras opens up new directions for developing protein degradation tools that are more controllable and safe. However, due to the potential for DNA damage from UVA radiation and its limited penetration into human tissues, the recent focus in this field is on finding and using more suitable light sources to achieve more precise control and safer PHOTAC designs.

#### 2.3.2 Semiconductor polymer nanoparticle SPNpro

SPNpro is a novel approach to address the off-target effects of PROTACs, developed by the Kanyi Pu team in 2021. SPNpro is a ternary complex composed of a semiconductor polymer core and cleavable peptides serving as cancer biomarkers, together with traditional PROTAC molecules (46). This complex possesses multiple functions: on one hand, when exposed to near-infrared light, SPNpro can generate efficient reactive oxygen species; on the other hand, it can induce a series of cancer immune responses, selectively killing tumor cells. During this process, SPNpro molecules can be cleaved by cancer biomarkers, such as cathepsin B, releasing traditional PROTAC molecules, thereby triggering the degradation of the target protein. SPNpro demonstrates high cancer tissue specificity and lower off-target effects, providing a new synergistic therapeutic approach for photodynamic therapy and protein degradation.

However, a drawback of SPNpro is its higher molecular weight, preventing its application through oral administration. Nevertheless, despite this limitation, research on SPNpro provides insights for developing more combined therapeutic strategies based on photodynamic therapy and protein degradation, offering new directions and opportunities for further exploration in the field of cancer treatment.

#### 2.3.3 Novel targeting chimeras

In recent years, to enhance the targeting efficacy of PROTACs and reduce off-target effects, there have been new targeting chimeras beyond those mentioned above. The design concepts for these targeting chimeras include connecting a folate group to traditional PROTAC molecules to develop float-PROTAC (47); antibody-based PROTACs (AbTACs); and ribonuclease-targeting chimeras (RIBOTACs); as well as transcription factor-PROTACs (TF-PROTACs) (48).

The emergence of these novel targeting chimeras aims to further enhance specificity and selectivity, thereby effectively guiding PROTACs to target proteins and reducing their impact on non-target proteins. For example, by introducing a folate group into PROTAC molecules, float-PROTAC can selectively recognize cancer cells through the highly specific binding of the folate receptor; antibody-PROTAC conjugates and antibody-based PROTACs (AbTACs) utilize high specificity and affinity of antibodies for selective target protein targeting and degradation (49); ribonuclease-targeting chimeras (RIBOTACs) utilize ribonuclease binding to target RNA sequences for target protein targeting and degradation (50); and transcription factor-PROTACs (TF-PROTACs) degrade target proteins by interfering with the function of transcription factors.

The emergence of these novel targeting chimeras provides new ideas and strategies for further optimizing the design of targeted drugs using PROTACs, with the potential to play a significant role in drug development and treatment. However, these targeting chimeras still need extensive experimental validation and clinical research to ensure their safety and effectiveness.

#### 2.3.4 Nanocarrier-delivered PROTAC

To overcome the limitations of targeted protein degradation, nanoparticle-mediated protein degraders (NanoPDs) have attracted considerable interest. Initially described 17 years ago, NanoPDs employ a variety of materials, degradation mechanisms, and linker chemistries to enable protein clearance via novel pathways.

These nanoparticle-based degradation platforms function through the specific conjugation of small molecules, peptides, or antibodies to achieve targeted functionality. Various strategies have been developed to attach functional moieties to nanoparticle surfaces, where the selection of conjugation method depends on both the ligand and nanoparticle type, while the choice of anchoring groups is influenced by the nanoparticle’s composition. The functionalization approach must account for reagent availability, potential byproducts, and the need for mild, scalable, and reproducible reaction conditions.

Early NanoPD systems primarily relied on lysosomal uptake and degradation as their mechanism of action. More recently, however, they have been engineered to engage the ubiquitin–proteasome system (UPS) by co-immobilizing ligands for both the protein of interest (POI) and E3 ligases on the nanoparticle surface, thereby promoting proteasomal degradation of the target protein (51).

Strategies utilizing lysosomal degradation take advantage of the inherent capacity of cells to transport nanoparticles into lysosomes. Upon binding of nanoparticle-mediated protein degraders (NanoPDs) to transmembrane proteins, cells internalize the nanoparticle–protein complexes via endocytosis. Subsequently, the NanoPD-containing endosomes fuse with lysosomes to form endolysosomal compartments. Within these structures, the nanoparticles function as lysosomotropic agents, necessitating only monofunctionalization with a ligand specific to the protein of interest (POI) (52).

In contrast to lysosome-based degradation approaches, strategies dependent on the ubiquitin–proteasome system (UPS)—such as PROTAC-based systems—require both a ligand that binds the protein of interest (POI) and a ligand that recruits an E3 ligase to facilitate intracellular target degradation (12).Compared to conventional small-molecule PROTACs, nanoparticles provide a distinct advantage by enabling the display of multiple copies of each ligand on their surface, leading to local concentration effects and enhanced multivalency (53).So far, only two E3 ligases—von Hippel-Lindau (VHL) and cereblon (CRBN), both belonging to the cullin-RING E3 ligase family—have been utilized in nanoparticle-based protein degraders (NanoPDs), mirroring their prevalence in small-molecule PROTAC strategies (54).

#### 2.3.5 Cereblon-based PROTACs

Cereblon-based PROTACs constitute the most extensively studied class of PROTAC molecules and have been the subject of intense research efforts in recent years for the treatment of a broad range of diseases, including cancer and neurological disorders. Several candidates have advanced into clinical trials, reinforcing their status as leading agents within the PROTAC domain. By engaging the Cereblon E3 ligase, these compounds have successfully mediated the targeted degradation of over 60 distinct proteins implicated in diverse pathologies such as cancer, neurological conditions, immune disorders, and viral infections—with several already showing encouraging clinical outcomes (55–57). To date, Cereblon-based PROTACs are the most widely reported in both scientific literature and patents, owing primarily to the favorable binding characteristics of Cereblon ligands compared to those recruiting other E3 ligases.

With respect to the synthesis of Cereblon-based PROTACs, three conventional strategies are commonly used to assemble the core components—the target-binding ligand, the linker, and the Cereblon ligand:

- Conjugation of the Cereblon ligand to the linker, followed by attachment to the target ligand;
- Conjugation of the linker to the target ligand first, followed by coupling with the Cereblon ligand;
- Separate attachment of two linker segments to the target ligand and the Cereblon ligand, followed by fusion of the two constructs.

In each strategy, the warheads (i.e., the target ligand and the Cereblon ligand) can be chemically modified to facilitate or optimize linkage to the connector. The choice of optimal synthetic route depends on several factors, including the availability and cost of building blocks, as well as the number of synthetic steps and purification processes required to obtain the final product (58).

#### 2.3.6 Targeted protein degradation technology via the lysosomal pathway

With recent advances in targeted protein degradation, several alternative strategies to proteolysis-targeting chimeras (PROTACs) have emerged that function independently of E3 ubiquitin ligase recruitment, greatly expanding the repertoire of targeted protein degradation. A number of new technologies primarily leveraging lysosomal mechanisms—such as AUTAC, AUTOTAC, ATTEC, LYTAC, MoDE-As, TransMoDE, IFLD, GlueTAC, KineTAC, TransTACs, AceTACs, and FRTAC—are being actively developed to target extracellular and membrane proteins. However, their applications remain at an early stage.

The lysosomal degradation pathway constitutes a fundamental proteolytic system independent of the proteasome (59–61), encompassing both autophagy and endosomal-lysosomal trafficking (62, 63). Building on this mechanism, Arimoto’s team developed the AUTAC system—a novel degradation technology that harnesses autophagy for targeted protein degradation. Although AUTAC is conceptually similar to PROTAC and also relies on ubiquitination, key mechanistic differences distinguish the two: while PROTAC recruits the protein of interest (POI) to an E3 ligase subunit to initiate K48-linked polyubiquitination, AUTAC promotes K63-linked polyubiquitination. This K63-linked ubiquitin signal is recognized by selective autophagy pathways, leading to degradation of the target POI (64). The AUTAC system uses modified guanine as a degradation tag, a warhead that binds the POI, and a flexible linker specific to the targe (60). Studies show that the synthetic guanine derivative p-fluorobenzyl guanine (FBnG) induces S-guanylation, which effectively promotes autophagic degradation.

Lysosome-mediated targeted protein degradation technologies overcome some limitations of proteasome-based degradation by exploiting autophagy and the endo-lysosomal pathway to selectively degrade pathogenic proteins and organelles, thereby broadening the range of druggable targets. Despite these promising mechanisms, each lysosome-directed TPD strategy faces specific challenges that require further investigation to optimize their application. Each targeted protein degradation modality offers distinctive advantages tailored to specific disease contexts, influenced by factors such as tissue-specific receptor expression and protein stability. Although still in early development, these innovative targeted protein degradation approaches hold significant potential to revolutionize the treatment of a wide range of diseases (65).

## 3 Clinical Applications of PROTACs

As of now, there are approximately over ten PROTACs globally in clinical development, and there are around 180 preclinical projects (**Table 1**). The following will provide an introduction with examples focusing on clinical Phase II drugs ARV-110, ARV-471, and Phase I drugs NX-2172, KT-474.

**Table 1:** List of PROTACs in Clinical Development.

Names	Structural formulas	Targets	E3 ligases	Indications	Status
ARV-471	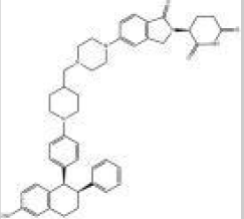	ER	CRBN	Breast cancer	Phase III
ARV-766	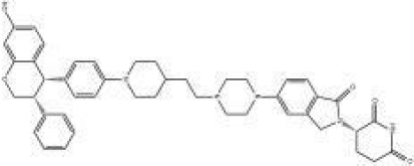	AR	CRBN	Prostate cancer	Phase III
ARV-110	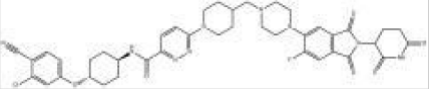	AR	CRBN	Prostate cancer	Phase II
CFT8634	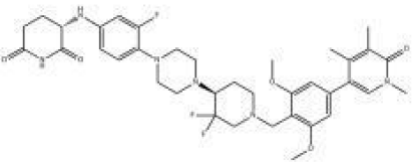	BRD9	CRBN	Synovial sarcoma	Phase I/II
AR-LDD	(undisclosed)	AR	CRBN	Prostate cancer	Phase I
GT20029	(undisclosed)	AR	Undisclosed	Androgenetic alopecia and acne	Phase I
NX-2127	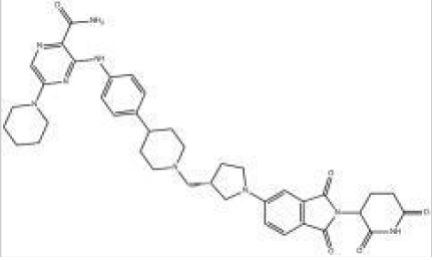	BTK	CRBN	B-cell malignancies	Phase I
HSK29116	(undisclosed)	BTK	Undisclosed	B-cell malignancies	Phase I
BGB-16673	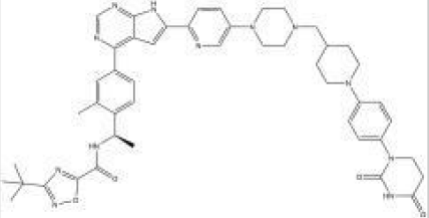	BTK	Undisclosed	B-cell malignancies	Phase I
DT-2216	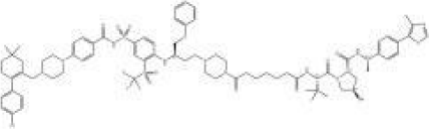	BCL-XL	VHL	Liquid and solid tumors	Phase I
FHD-609	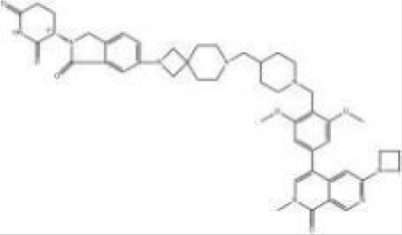	BRD9	Undisclosed	Synovial sarcoma	Phase I
LNK01002	(undisclosed)	RAS GTPase	Undisclosed	Acute myeloid leukemia	Phase I
KT-474	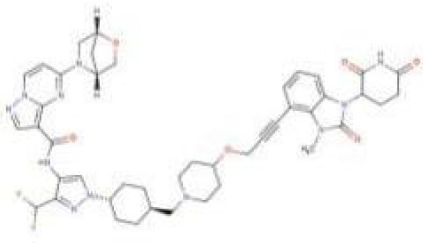	IRAK4	Undisclosed	Atopic dermatitis and hidradenitis suppurativa	Phase I
KT-413	(undisclosed)	IRAK4	CRBN	B-cell 1ymphomas	Phase I
NX-5948	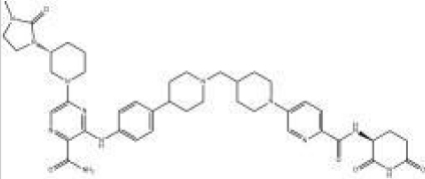	BTK	CRBN	B-cell malignancies and autoimmune diseases	Phase I
KT-333	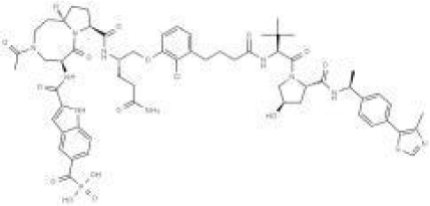	STAT3	Undisclosed	Liquid and solid tumors	Phase I
CG001419	(undisclosed)	TRK	CRBN	Cancer and other diseases	IND-e
CFT8919	(undisclosed)	EGFR	CRBN	Non-small cel1 lung cancer	IND-e
HHP-518	(undisclosed)	AR	CRBN	Prostate cancer	Phase I

### 3.1 Phase II clinical drug

#### 3.1.1 ARV-110

**Figure 3:**
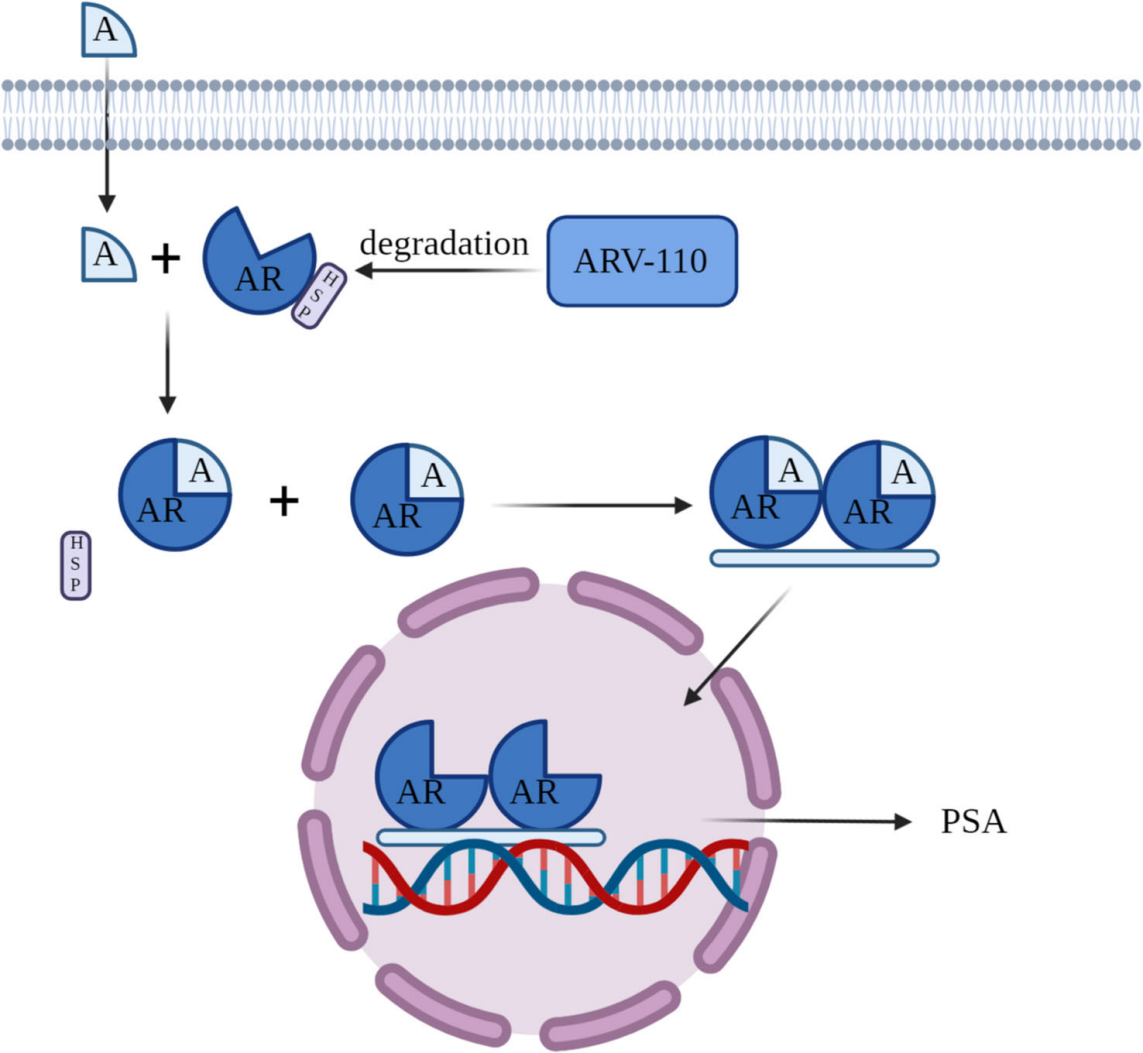
Mechanism of Action of ARV-110.

ARV-110 is an orally bioavailable experimental PROTAC (PROteolysis TAgeting Chimeras) protein degrader designed for the selective degradation of the androgen receptor (AR). The AR gene encodes the androgen receptor protein, with testosterone and dihydrotestosterone as its primary ligands. This receptor is extensively found across a majority of organs and tissues in the human body. Within the cytoplasm, the androgen receptor attaches to heat shock proteins (Hsp). When androgens bind to the androgen receptor, it becomes activated, resulting in the separation of heat shock proteins. The androgen receptor subsequently dimerizes, moves into the cell nucleus, and attaches to androgen response elements (ARE) on DNA, triggering the transcription of downstream genes, such as prostate-specific antigen (PSA). This process promotes tumor growth (**Figure 3**). ARV-110 employs these previously mentioned principles via PROTAC technology to break down the AR protein and has been officially advanced as a prospective therapy for metastatic castration-resistant prostate cancer (mCRPC). The development of this drug represents the potential of PROTAC technology in the field of prostate cancer treatment, offering new therapeutic hope for patients with mCRPC.

Arvinas announced data from Phase I and Phase II clinical studies of ARV-110 at the ASCO-GU conference on February 14, 2022 (NCT03888612) (66). The findings confirm multiple clinical benefits of ARV-110 in the treatment of patients with metastatic castration-resistant prostate cancer (mCRPC). Firstly, ARV-110 demonstrates durable and reliable therapeutic effects. In patients with tumors harboring the ART878X/H875Y mutation, the response rates for prostate-specific antigen (PSA) reduction by 50% (PSA50) and 30% (PSA30) were 46% and 57% (67), respectively. Additionally, among the evaluated patients with ART878X/H875Y mutations, two experienced sustained symptom relief, and six achieved tumor reduction. Even in tumors without the ART878X/H875Y mutation, decreases in PSA levels and tumor shrinkage were observed, indicating a broader potential for ARV-110 in prostate cancer treatment. Secondly, ARV-110 demonstrates good safety. In an experiment assessing patients’ tolerance to adverse reactions, 113 patients were treated with the recommended Phase II dose (RP2D) of 420mg. The majority of patients experienced grade 1–2 treatment-related adverse events (TRAE) (68), such as nausea, fatigue, and vomiting. No TRAE of grade ≥4 was reported, and few patients had to reduce or discontinue the dose due to TRAE, highlighting the drug’s favorable safety profile, which is crucial in the context of everyday pharmacotherapy.

However, despite positive early data, Arvinas pivoted in 2023 to advance its second-generation PROTAC drug ARV-766 and initiated a Phase III clinical trial in 2024. This decision was primarily based on several core reasons across key dimensions such as efficacy scope, mutation coverage, and tolerability.

The coverage ability of ARV-110 against drug-resistant mutations, especially L702H, is insufficient. ARV-110 can degrade wild-type AR and some mutations, such as T878X/H875Y, but cannot degrade AR L702H mutation. This mutation accounts for approximately 11% of mCRPC patients receiving novel hormone therapies such as enzalutamide or abiraterone, and is one of the main mechanisms leading to drug resistance. Clinical data shows that only 8% of patients with L702H mutation who receive ARV-110 treatment achieve PSA50, indicating significantly limited efficacy. As a second-generation PROTAC, ARV-766 achieved effective degradation of L702H mutation through structural optimization, such as replacing CRBN ligand with benzamide and adjusting linker ring system. In clinical trials, patients carrying the L702H mutation had a PSA50 rate of 50%, expanding the coverage population to three times that of ARV-110.

ARV-766 has a wider range of clinical efficacy and patient benefit groups, while the efficacy of ARV-110 is concentrated in specific subgroups. In T878X/H875Y mutation patients without L702H co mutation, the PSA50 rate of ARV-110 was 54%, and rPFS reached 11.1 months; But if there is a L702H mutation, the therapeutic effect is significantly reduced. On the other hand, ARV-766 has the advantage of full mutation spectrum coverage. The PSA50 rate for all AR LBD mutation patients is 41%; It is effective for both single and compound mutations of L702H, and has a higher response rate for patients with T878/H875 co mutations; The tumor suppression effect is more long-lasting, and some patients have observed confirmed tumor shrinkage.

The structural optimization of ARV-766 has improved tolerance and drug properties. It achieves comprehensive performance superior to ARV-110 through three key structural modifications: linker optimization: changing the hexagonal ring to a quaternary ring to enhance the binding stability to mutant AR; AR ligand modification: Replace chlorine atoms with methoxy groups to increase exposure and tissue permeability; CRBN ligand upgrade: Replace thalidomide with benzamide to avoid degradation of non target proteins (such as IKZF1/3, GSPT1) and reduce the risk of blood toxicity; Enhance plasma stability and reduce side effects caused by metabolites. Clinical results have confirmed that ARV-766 has better tolerability: the incidence of treatment-related adverse events (TRAE) ≥ 3 is low, as the TRAE discontinuation rate is only 4%.

Although ARV-110 has validated the conceptual feasibility of PROTAC in prostate cancer, its ineffectiveness against the key drug-resistant mutation L702H limits its clinical value. ARV-766 has achieved extensive mutation degradation ability, better safety, and wider patient coverage through structural innovation, becoming a better solution to solve the problem of mCRPC resistance. This shift reflects the R&D logic of “iterative upgrading” in the PROTAC field, and highlights the necessity of precise treatment strategies based on biomarkers in advanced cancer.

A direct comparison of the core characteristics between PROTACs ARV-110 and ARV-766 is presented in **Table 2**.

**Table 2:** Comparison of the core characteristics between ARV-110 and ARV-766.

Characteristics	ARV-110	ARV-766
L702H mutation degradation	Ineffective(PSA50 8%)	Effective(PSA 50%)
Other mutations PSA50	36%-54%	41%(full mutation spectrum)
Tolerability	controllable, but fatigue and nausea are common	better. only 4%discontinuation
Proportion of patients covered	limited	3 times higher than ARV-110
R&D status	Phase II suspended	phase III in progress

#### 3.1.2 ARV-471

ARV-471 is clinical oral bioavailable PROTAC (PROteolysis Targeting Chimeras) protein degrader to degrade estrogen receptor (ER) specifically and treat some ER+/HER- metastatic breast cancer patients.The ER can induce more co-regulatory elements to bind to target proteins such as heparin binding epidermal growth factor, epidermal growth factor receptor, human epidermal growth factor receptor, insulin like growth factor 1 receptor quickly activate the estrogen signaling pathway, transcription of cell cycle gene that related with oncogenesis, promote breast cancer cells proliferate very effectively.

ARV-471, as single agent or combined with CDK4/6 inhibitor, can also show superior anti-tumor effect with more value than common breast cancer drugs like fulvestrant and palbociclib. Early phase clinical evidence shows that ARV-471 shows almost 100% degradation capability of ER in the tumor cell, and ARV-471 could induce great shrinkage of cancer cell as a single agent in Xenograft models with multiple ERs, revealed clear and good clinical benefit signal in a great number of pretreated patients. As an experiment group in combination with palbociclib, the tumor is reduced to 31%, and the tumor size in a group of 10 mice decreased 80%. In clinic, showed great anti-tumor value as well.

In the VERITAC trial (NCT04072952) (69), In total 71 patients were given the treatment with ARV-471, all of these patients received pretreatment with CDK4/6 inhibitors,79% of them had ever been pretreated by fulvestrant, and 73% of them had received chemotherapy. ARV-471 demonstrated good tolerability at doses of 200 mg once daily (QD) or 500 mg QD, with most treatment-related adverse events (TRAE) being grade 1-2, primarily nausea, fatigue, and vomiting. Incidences of dose reduction and discontinuation due to TRAE were less than 2%. Additionally, ARV-471 showed high efficacy. In 47 evaluable cases of clinical treatment benefit, the clinical benefit rate (CBR) was 40%.as of the date of data cut, the number of patients continuously received study treatment is 14, two of them have been continuously received study treatment more than 18 months. In term of degradation level of ARV-471 in tumor cell, in dosing of 500mg, ARV-471 could degrade the ER more than 90%, the median was 6%, average was 64%, compared to data 40%-50% were reported previously (70).

Need mention, compared to small molecule drug, because PROTACs own relatively high molecular weights, such as: 0.7-1. kDa, and multiple hydrogen bond donors, high lipid solubility, larger polar surface. All these factors cause PROTAC drug low permeability, and poor solubility, results in poor oral bioavailability of PROTAC drug. Some reports showed, PROTACs can improve the drug solubility, especially intestinal fluid solubility after a fed meal (71), maybe higher exposure of drug in intestinal fluid can cause this. That we can see in dosing regimens of “with meals once per day” design of early clinical trials of ARV-110 and ARV-471.

### 3.2 Phase I clinical drug

#### 3.2.1 NX-2127

**Figure 4:**
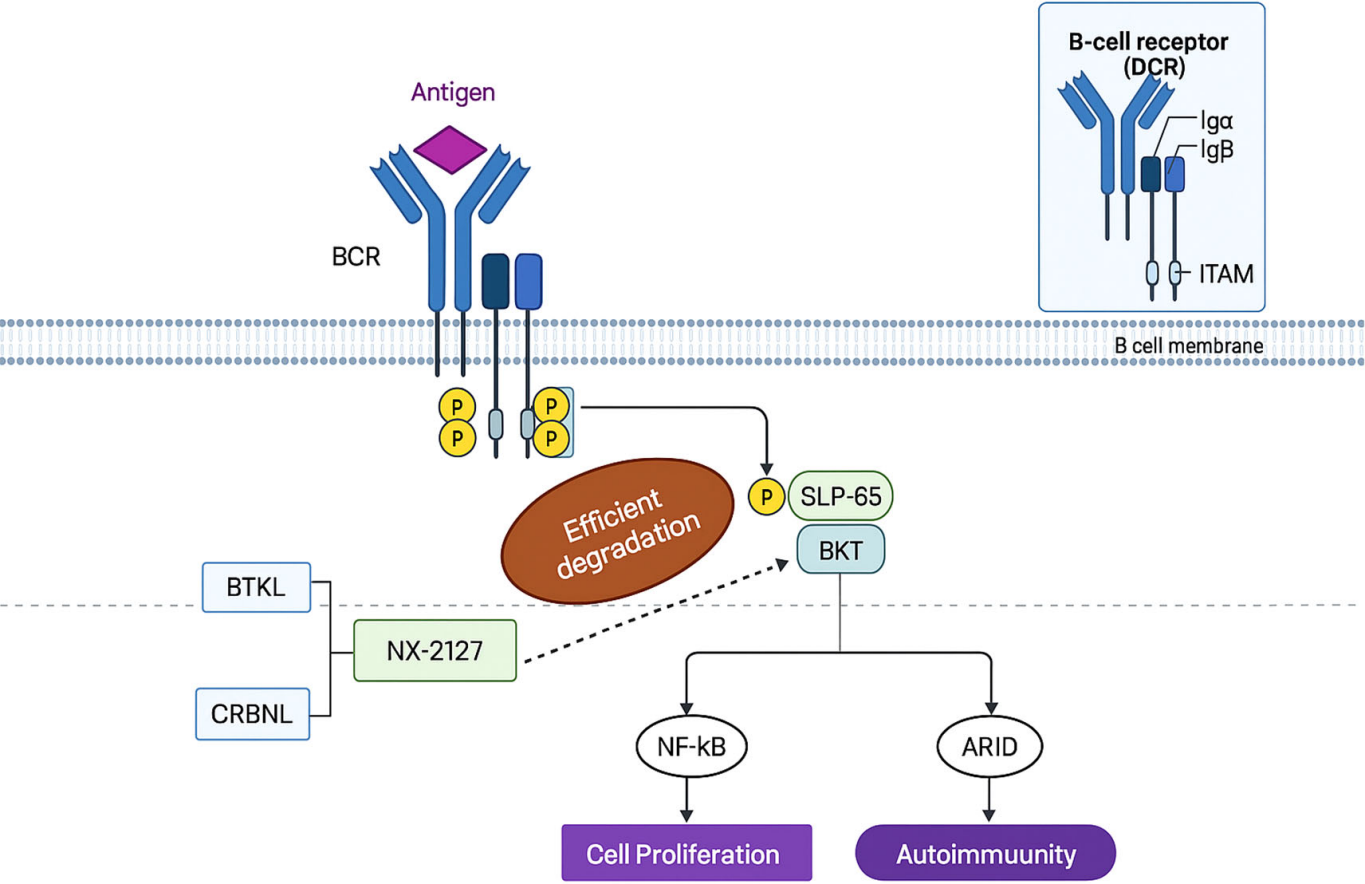
The principle of NX-2127.

NX-2127 is a bifunctional proteolysis-targeting chimera (PROTAC) comprising a Bruton’s tyrosine kinase (BTK)-binding ligand and a cereblon (CRBN)-recruiting moiety. This agent hijacks the CRBN-dependent ubiquitin-proteasome system to induce BTK degradation—a pivotal regulator of B-cell activity whose inhibition ameliorates B-cell malignancies (e.g., chronic lymphocytic leukemia [CLL]) and autoimmune disorders (e.g., graft-versus-host disease) (**Figure 4**).

Although clinically approved BTK inhibitors face limitations due to intolerance or resistance mutations (e.g., BTK-C481S), IKZF3 overexpression drives resistance in CLL (72). NX-2127 simultaneously degrades BTK and IKZF proteins (mediated by its CRBN ligand), thereby circumventing resistance mechanisms and enhancing antitumor efficacy. Consistently, NX-2127 achieves potent BTK degradation and suppresses proliferation in TMD8 cells, outperforming conventional BTK inhibitors. Critically, it overcomes BTK-C481S-mediated resistance in mutant cell lines, offering a promising therapeutic strategy for patients with refractory disease.

NX-2127 demonstrates promising clinical activity in relapsed/refractory B-cell malignancies. The Bruton’s tyrosine kinase (BTK) degrader NX-2127 (Nurix Therapeutics, Inc.) has shown positive results in a Phase I clinical trial (NCT04830137) (73). In heavily pretreated relapsed/refractory chronic lymphocytic leukemia (CLL) patients, NX-2127 achieved >90% sustained BTK degradation with clinically meaningful responses in 67% (4/6) of patients, including partial responses in those harboring BTK-C481S mutations.

Initial Phase Ia/Ib data (October 2021) from 6 BTK inhibitor-refractory patients demonstrated robust BTK reduction (median 94%, P<0.01 vs. baseline) and universal clinical activity in the 100 mg cohort (3/3 responses), alongside a favorable safety profile with no treatment-related deaths, SAEs, or DLTs.

Updated results (ASH 2023) confirmed dose-dependent pharmacokinetics (mean t~1/2~=3.2 days) and durable responses (>18-month PFS in one patient).

Dose-limiting toxicities (DLTs) occurred in two patients:

- One CLL patient developed cognitive impairment;
- One marginal zone lymphoma (MZL) patient experienced Grade 4 neutropenia.

Both DLTs emerged in the 300 mg cohort.

Treatment-related adverse events (TRAEs) included:

- Most common TRAEs (all grades): fatigue (48.9%), neutropenia (42.6%), hypertension (36.2%);
- Grade ≥3 TRAEs: neutropenia (38.3%), hypertension (14.9%), anemia (12.8%);
- Other notable events: contusion (27.7%, all Grade <3) and atrial fibrillation (12.8%, including 6.4% Grade ≥3).

Among patients discontinuing NX-2127, the primary reasons were:

- Progressive disease (PD) (25.5%);
- Adverse events (AEs) (21.3%).

With a median follow-up of 9.5 months (range: 0.1–24.3), NX-2127 exhibited a manageable safety profile, consistent with prior reports on BTK-targeted and immunomodulatory therapies. Importantly, NX-2127 elicited encouragingly durable responses in this heavily pretreated population, including patients with BTK resistance mutations in non-Hodgkin lymphomas (NHL) and chronic lymphocytic leukemia (CLL) (74).

On March 11, 2024, Nurix Therapeutics announced the restart of its Phase I trial (NCT04830137) with an optimized manufacturing process, prioritizing enrollment of aggressive non-Hodgkin lymphoma (NHL) patients with documented therapy resistance—including diffuse large B-cell lymphoma (DLBCL) and mantle cell lymphoma (MCL)—where primary endpoints encompass dose-limiting toxicities (DLTs), maximum tolerated dose (MTD), objective response rate (ORR) per Lugano criteria, and incidence of adverse events (AEs)/laboratory abnormalities, while secondary endpoints comprise pharmacokinetics (PK), duration of response (DOR), progression-free survival (PFS), overall survival (OS), and complete response (CR) rate, with study completion projected by December 2025.

#### 3.2.2 KT-474

KT-474 is a potential first-in-class oral PROTAC developed by Kymera Therapeutics. It functions by binding to IRAK4 and recruiting E3 ubiquitin ligases to label IRAK4 with ubiquitin. These labels guide the IRAK4 protein to be degraded by the cellular “garbage disposal system” proteasome, thereby blocking IRAK4-mediated signaling. In comparison to antibody therapies targeting cytokines that activate Toll-like receptors (TLRs) and IL-1 receptors, targeting IRAK4 can simultaneously block signaling pathways of various inflammatory cytokines. Additionally, by degrading IRAK4, KT-474 can also disrupt its role in structural protein-mediated signaling, achieving effects that IRAK4 kinase inhibitors alone cannot achieve.

In the Phase I clinical trial of KT-474 (NCT04772885) (75), researchers divided volunteers into seven groups for a Single Ascending Dose (SAD) study, starting with a dose of 25mg and gradually increasing, with 8 volunteers in each group, including 2 receiving a placebo. Currently, dosing has been completed for four groups, with doses of 25mg, 75mg, 150mg, and 300mg. No treatment-related adverse reactions were observed in the 24 volunteers who received the drug, indicating a low off-target effect of the protein degrader KT-474 itself. In the 300mg dose group, the median degradation rate of IRAK4 was 90% (average 84%).

To assess the biological impact of lowering IRAK levels, researchers extracted blood samples from volunteers after KT-474 treatment. They stimulated cytokine production using activators of Toll-like receptors (TLRs) *in vitro* and measured the levels of various cytokines. The results showed that, 24–48 hours after KT-474 treatment, the two highest doses significantly reduced the levels of various pro-inflammatory cytokines induced by TLR agonists, with a maximum reduction of up to 97%.

In terms of safety, KT-474 demonstrated good safety and tolerability. The most common adverse events were mild or moderate headaches or nausea, and no severe adverse events occurred. The single-dose mechanism and biological mechanism of KT-474 validate the drug’s efficacy. Furthermore, it confirms that targeting the degradation of IRAK4 is an effective strategy to maximally block the signaling of Toll-like receptors (TLR) and interleukin-1 receptors (IL-IR).

On November 13, 2023, Kymera announced additional Phase I clinical trial data for KT-474 (SAR444656). These results further confirm the drug’s positive impact on alleviating disease burden and symptoms in patients with hidradenitis suppurativa (HS) and atopic dermatitis (AD), with systemic anti-inflammatory effects observed in moderate-to-severe cases (36).

Notably, KT-474 has dosed the first patient in its Phase II clinical trial for atopic dermatitis (AD), marking a critical advancement in its non-oncology applications.

## 4 Challenges in PROTAC development

Over the past two decades, PROTAC technology has rapidly evolved into a cutting-edge therapeutic modality. Recent positive Phase I/II clinical trial outcomes demonstrate considerable potential for treating cancers and other diseases. As an innovative therapeutic strategy, PROTAC offers distinct advantages unattainable with conventional drugs. Initial clinical candidates targeted clinically validated proteins, enabling assessment of *in vivo* safety and efficacy while establishing PROTAC’s utility for solid tumors. Crucially, PROTAC’s transformative potential lies in targeting “undruggable” proteins – a mechanism fundamentally distinct from traditional small-molecule inhibition that unlocks novel therapeutic avenues (76). Continued technological refinement promises expanded applications across diverse diseases.

Despite these advances, PROTAC development faces significant challenges. Currently, no established “gold standard” analogous to Lipinski’s Rule of Five exists for target selection. An ideal PROTAC target would need to meet the following criteria:

1. Pathogenic Alteration: The target should display a pathogenic gain-of-function alteration, such as overexpression, mutation, or mislocalization (77).
2. Ligand-Binding Pocket: It must possess a ligand-binding pocket for PROTAC molecule binding (78).
3. Ubiquitination Site: The target surface should feature an accessible site for E3 ubiquitin ligase binding (79).
4. Structure: The target requires a flexible structure susceptible to degradation by the proteasome (80).

### 4.1 Target protein (Protein of interest)

PROTACs currently under investigation have demonstrated effective binding to the POI in numerous *in vitro* and *in vivo* studies. Importantly, high-affinity or covalent binding between the POI and the PROTAC warhead is not a prerequisite for efficacy. Research on the multi-kinase inhibitor foretinib revealed that PROTACs incorporating this warhead degraded only a small subset of relevant kinases. The overall success of kinase degradation hinges primarily on the extent of POI-PROTAC-E3 ubiquitin ligase ternary complex formation, rather than on the intrinsic binding strength of the warhead to its target protein. In fact, high-affinity or covalent POI-warhead binding can impede PROTAC dissociation from the ternary complex, a process critical for sustained protein degradation (81). Such excessive binding may promote an occupancy-driven mechanism over an event-driven mechanism, thus diminishing the unique benefits of PROTACs.

Ongoing PROTAC research focuses on optimizing both the target protein engagement and the warhead, potentially yielding novel effective therapies. While no perfect method for PROTAC target selection currently exists, investigating ternary complex formation and optimal ligand selection holds promise for overcoming challenges in targeting difficult-to-proteins (82).

### 4.2 Oral bioavailability of PROTACs requires improvement

By traditional small-molecule standards, PROTACs are considerably larger. Integrating the three components into a single molecular unit confers advantages but typically yields structures of 300–500 Da; PROTACs themselves generally range from 700 to 1000 Da. This substantial size poses significant challenges for oral administration. Current administration routes commonly include intraperitoneal, subcutaneous, or intravenous injection. Intravenous delivery circumvents the gastrointestinal absorption phase required for oral drugs and is compatible with established PROTAC formulations. Subcutaneous administration has gained considerable interest due to its potential for sustained effects, reducing dosing frequency (83).

However, both parenteral methods exhibit limitations. For example, subcutaneous administration often faces tolerability challenges, where even approved drugs utilizing standard formulation components can cause injection site pain and granulomas (84). Consequently, oral administration remains the preferred route for small-molecule drugs, offering simplicity, broad acceptance, and preserved drug exposure. Oral formulations also enable strategies to enhance the delivery of poorly soluble compounds. Theoretically, absorption of oral PROTACs may be improved by incorporating permeability enhancers and efflux inhibitors into their formulations. A notable drawback, however, is that oral administration complicates therapeutic monitoring.

Significant efforts are underway to enhance PROTAC oral bioavailability. Determining appropriate dosing regimens for PROTACs presents greater complexity than for traditional inhibitors. Excess PROTAC concentrations can drive the formation of binary complexes (target-PROTAC and E3-PROTAC) rather than the desired ternary complex. Critically, ternary complex formation is precluded if either the target protein or E3 ligase is present in substantial stoichiometric excess relative to the PROTAC (83). *In vivo* animal studies demonstrate that all evaluated compounds exhibit plasma half-lives exceeding 4 hours. Notably, RPROTAC 33c (TD-802) displays the longest half-life at 8.28 hours. However, these PROTACs suffer from poor oral absorption.

To address this limitation, Tuckwell and colleagues developed AR-targeting PROTACs engaging CRBN, demonstrating stability in mouse liver microsomes (85). Additionally, Pack’s team found that plasma protein binding strongly correlates with higher lipophilicity; consequently, PROTACs with elevated ChromLogD values exhibit high plasma binding (>99%). Their research also identified key metabolic hotspots in PROTACs, including the inherently labile glutarimide moiety of thalidomide-derived ligands and linker functional groups (e.g., amides), where hydrolysis contributes to increased clearance. Prodrug design offers a promising approach to enhance bioavailability. For instance, in 2021, Wei and colleagues developed a prodrug strategy incorporating lipophilic groups onto the CRBN ligand. This approach successfully improved oral bioavailability to 68% (86) and concurrently demonstrated tumor shrinkage efficacy in mouse models (87).

### 4.3 Target limitations

PROTACs exhibit inherent limitations in degrading specific protein classes. Integral transmembrane proteins lacking significant cytoplasmic exposure present a fundamental challenge: their capture is difficult, rendering subsequent ubiquitin delivery for proteasomal degradation impossible. Consequently, such proteins are generally unsuitable PROTAC targets. Degradation of proteins with expanded repeat sequences or aggregation-prone tendencies also proves particularly challenging.

Research suggests autophagy may provide a more effective pathway than PROTACs for degrading aggregation-prone proteins. Consequently, several companies are developing degraders leveraging lysosomal and autophagic pathways. Lysosome-Targeting Chimeras (LYTACs) achieve degradation by coupling a target-specific antibody to glycans that trigger lysosomal degradation (88). Similarly, Autophagy-Targeting Chimeras (AUTACs) connect a target ligand to a degradation tag to engage the autophagy machinery (89).

Emerging start-up companies are now exploring non-ubiquitin-proteasome system degradation strategies to address specific targets. Notably, the proteasome cannot degrade organelles such as mitochondria, peroxisomes, or lipid droplets. Despite these constraints, PROTACs retain a broad target range and substantial market potential.

### 4.4 Off-target effects and systemic toxicity

Similar to other targeted therapies, prolonged PROTAC exposure can induce cellular adaptation or mutations in the target protein or E3 ligase, diminishing degradation efficiency over time and potentially conferring drug resistance. Furthermore, the inherent complexity of PROTAC molecules and their intracellular mechanisms may prompt cancer cells and other disease-relevant systems to activate compensatory pathways or evasion strategies.

Off-target effects and systemic toxicity remain primary safety concerns; their high potency and irreversible degradation mechanism mean unintended interactions with non-target proteins could trigger unpredictable biological outcomes or adverse effects. A key challenge is the current absence of standardized validation protocols for assessing on-target versus off-target activity, selectivity, and proteasome dependency.

Beyond biological challenges, PROTACs’ synthetic complexity poses significant hurdles. It substantially escalates production costs and extends development timelines, as each synthesis step requires optimization and intermediate purification is often labor-intensive. Pharmaceutical formulation, particularly developing stable solid forms suitable for clinical use, introduces further difficulties (76).

In summary, while PROTAC technology holds significant promise, advancing it necessitates addressing multiple limitations—from suboptimal pharmacokinetics and bioavailability to toxicity, resistance mechanisms, and manufacturing obstacles. Addressing these issues will require continued innovation in novel drug design, delivery systems, and validation standards (90).

### 4.5 Limitations of E3 ligases

Significant gaps remain in understanding how PROTACs traverse the cell membrane. Further research is required to elucidate their uptake, diffusion, metabolism, excretion, and toxicity. Enhancing cellular uptake and bioavailability remains a key challenge for achieving and maintaining PROTAC concentrations necessary for pharmacological efficacy (83). Molecules with optimal physicochemical properties are therefore essential to overcome these hurdles. The susceptibility of the target protein FKBP12 to degradation by the compound RC32 highlights the potential of optimizing PROTACs with suitable linkers to improve permeability (91).

While over 600 E3 ligases, all critical for proteasome-mediated protein degradation, have been identified in humans, only a small subset possess known small-molecule ligands (92). Notably, the majority of published PROTACs (Proteolysis Targeting Chimeras) rely predominantly on just one E3 ubiquitin ligase, presenting a major obstacle to broadening the technology’s applicability. Consequently, researchers are actively pursuing more specialized E3 ligases (93) and ligands targetable to specific cells or tissues. Promising recent progress includes the discovery of ligands recruiting the aryl hydrocarbon receptor (AhR) E3 ligase (94).PROTACs selectively ubiquitinate their target protein only upon forming a stable ternary complex with an E3 ubiquitin ligase. However, progress in understanding PROTAC-mediated ternary complexes is currently limited by the technical challenges associated with detecting these complexes and obtaining their crystal structures (95).

## 5 Summary and outlook

Drug discovery continues to evolve dynamically. PROTACs represent a highly promising therapeutic strategy, leveraging unique mechanisms to target traditionally undruggable proteins. Having advanced over two decades from concept to clinical investigation, PROTACs now feature over ten candidates in active trials. Their event-driven pharmacology circumvents fundamental limitations of occupancy-driven inhibitors.

Significant challenges persist in PROTAC development and deployment. Clinical success necessitates multidisciplinary convergence across medicinal chemistry, nanotechnology, pharmaceutical sciences, and clinical research.

By optimizing strategies to enhance solubility, improve tumor selectivity, and reduce off-target toxicity, these engineered degraders may fulfill their transformative potential in oncology and complex disease therapeutics. As the technology matures, PROTACs are positioned to emerge as a major therapeutic modality alongside established approaches including small-molecule inhibitors, monoclonal antibodies, and immunotherapies.

Sustained collaboration in discovery, development, and clinical translation, augmented by pharmaceutically validated excipients, will enable PROTACs and related platforms to redefine disease treatment paradigms in the coming decades (96).
